# Alternation of Remedies

**Published:** 1887-03

**Authors:** P. P. Wells

**Affiliations:** Brooklyn, N. Y.


### HOMŒOPATHIC PHYSICIAN,

A MONTHLY JOURNAL OF MEDICAL SCIENCE.

"If our school ever gives up the strict inductive method of Hahnemann, we are lost, and deserve only to be mentioned as a caricature in the history of medicine."—CONSTANTINE HERING.

Vol. VII. MARCH, 1887. No. 3.

### ALTERNATION OF REMEDIES.

P. P. WELLS, M. D., BROOKLYN, N. Y.

But is it never allowable to give two remedies in alternation? No doubt it is, but, almost if not quite, never in the treatment of this fever [typhoid].—*Myself* in *American Homœopathic Review*, Vol. III, p. 349, 1863.

"Doctor, before you die, put yourself right on the record as to this matter of alternation, and not leave this to be called up as a justification of error, wrongfully, as are sayings of Hahnemann, Hering, Dunham, and others, after their death, when they can no longer protect themselves."—*My Friend.*

It may not be amiss in attempting compliance with the request of my friend to note the time (1863) and circumstances in which the above statement of the sometimes admissibility of alternation was written.

When I began endeavors to practice homœopathically, almost all whom I knew as homœopathic physicians gave *two*, and some of them even *more*, remedies *at the same time*, to be given in alternation at short, specified intervals, without reference to, or a knowledge of, what would be the phenomena of the case at the specified time, when any dose of either was to be given. The doses were to be repeated without knowing whether either or neither of the medicines would then be *like the phenomena of the case under treatment.* Up to the time when the sentence above was written, this was so generally true of these doctors that one of them (the late Dr. J. R. Cox, of Philadelphia), on page 362 of the third volume of the *Review*, says: "The opponents of alternation being not, as I believe, more than five or six per cent. of the whole number of homœopathic practitioners throughout the civilized world."

So great had been the force of example of so great numbers, that new recruits to the ranks of converts to our school fell, as I did, into this senseless habit of practice for no other or better reason than this—that others were, so many of them, doing the same thing. It was assumed that what was so general must be right. And because so general, there must be, somewhere, a *scientific* foundation for the practice. I had promised myself to find this foundation, and spent time and thought in seeking it, but, because there *is* no such foundation, the search was fruitless, except that it led ultimately to the clear view of the fact that the habit was altogether *unscientific*, and wholly without reason or excuse.

It was while engaged in this search for a non-existent scientific basis for this so general practice, that the sentence at the beginning of this paper was written. I had progressed so far as to see clearly that alternation had no place in a right application of homœopathic law and means to treatment of typhoid fever. This I had fully stated, but so great was the force of the impression, that what so many earnest and honest men, who had been before me in the practice were doing, must, somehow and sometimes, be right, and, if right, then it must have foundation in philosophic principles, that I followed this statement with the objectionable sentence above, that alternation was *sometimes* allowable.

Then the questions would rise, What are these times, and what are the principles which justify this practice at these times? I set myself to answer these questions with full confidence that I should be able to do this satisfactorily to myself. But the more I studied for answers to these questions the more they confirmed themselves to my mind as insoluble puzzles. Insoluble because there *are* in the very nature of things *no such times* nor *principles* to be discovered. And because there are neither, neither have been discovered by any man. The sentence above was written just as or but little before the writer had emerged into a clear conviction of this truth. He sought to find out the cases proper for alternation, and the reasons which made them subjects for this, and the result has been an unhesitating assurance that neither cases nor reasons have any existence whatever, other than in the imaginations of erring, though perhaps honest, men.

So great was my confidence at the time of writing this sentence now objected to as above, that there *must* be some principles to justify this practice, at least in some cases, that in the outset of my search for these I thought I saw a *class* of such cases, and wrote in the same paragraph which contains this which my friend feared would disgrace me after my death:

"The kind of case is that where the characteristics of the disease do not find their simillimum in those of any one known drug, but in those of *two* cognate drugs they are found. So there is *law* for the government of alternations of drugs, and that this is nothing but the same old law of similars which rightly controls all drug selections."

This seemed, at the time it was written, to be a satisfactory answer to the question as to the *kind* of cases in which drugs should be alternated. It is certainly plausible, and has deceived many to their hurt. It is only plausible, as appears most clearly when brought to the light of the law it would give for its justification. This law says *the most similar*, not *the most similars*. Now, as there are no two drugs identical in their action on the living forces, the idea of *two* "most similar" in their effects is a demonstrated absurdity. The differences between the two will show *one* of them to be the "most similar" to the case to be treated, if either of them is, and it is this *one* which the law demands for a cure, and not the two. This *one*, the "most similar," being found and given, the law promises a cure. It never has promised this to any *two*, given alternately, at short intervals, in the absence of all knowledge as to whether either of them will be similar at all to those elements of the sickness a likeness to which in the remedy the law demands to constitute it a curative in any case. This plausibility, we have said, has deceived many. I have been one of them. This deception has been carried so far with some practitioners that they have, and logically, gone so far as to say:

"The conscientious homœopath finds himself unable to fully satisfy his mind as to a single remedy. * * * Two, three, four occur to his mind, but he is unable to distinguish conclusively between them. What shall he do? * * * Give all four *at the same time*, and the prescriber *is criminal if he does not*."* [Italics the Doctor's.]—*W. S. Searle, M. D., in N. A. Journal of Hom., May*, 1882.

Perhaps absurdity in professional writing has never gone further. And yet if *two* drugs may be lawfully given in alternation, why not four? This writer probably considers himself

---

* The fault here is not with the "conscience" nor the logic of the writer, but with his *premises*. The law says the "most *similar*" remedy. This writer says he can't find it or does not know it when he sees it, and this he makes a sufficient reason for himself or any other man, for abrogation of God's law, and says whoever will not do this "*is criminal*." False premise and ignorance, "that is what is the matter." Crime is a *violation of law*, not a refusal nor neglect of this violation.

a "conscientious *homœopath*," though the law says *one* remedy, while he says *four* " *at the same time.*" Is it not evident that though he may be very "conscientious," he is *no* homœopath, unless one may be so considered while he sets aside the fundamental principles of Homœopathy whenever his very limited knowledge of its materia medica leaves him in doubt as to which of any *four* drugs is most similar in its pathogenesis to the phenomena of his case?

Since the obnoxious sentence which might have disgraced me was written, now many years ago, alternation has ceased to be a part of my professional acts. I soon after found that in the light of the philosophy of Homœopathy this practical error could never be allowable, and my practice has since been strictly in accordance with this conviction. I look for *one* remedy, and not for *two*, and when this is found and given, the results are greatly more satisfactory to myself and beneficial to my patients.

Am I now "*right on the record*"?

